# Modulation of Human Immune Responses by Bovine Interleukin-10

**DOI:** 10.1371/journal.pone.0018188

**Published:** 2011-03-25

**Authors:** Gerco den Hartog, Huub F. J. Savelkoul, Ruud Schoemaker, Edwin Tijhaar, Adrie H. Westphal, Talitha de Ruiter, Elise van de Weg-Schrijver, R. J. Joost van Neerven

**Affiliations:** 1 Cell Biology and Immunology Group, Wageningen University, Wageningen, The Netherlands; 2 Royal FrieslandCampina Research, Deventer, The Netherlands; 3 Laboratory of Biochemistry, Wageningen University, Wageningen, The Netherlands; WIV-Pasteur Institute, Belgium

## Abstract

Cytokines can be functionally active across species barriers. Bovine IL-10 has an amino acid sequence identity with human IL-10 of 76.8%. Therefore, the aim of this study was to evaluate whether bovine IL-10 has immunomodulatory activities on human monocytes and dendritic cells. Peripheral blood monocytes were isolated from healthy donors, and used directly or allowed to differentiate to dendritic cells under the influence of IL-4 and GM-CSF. Recombinant bovine IL-10 inhibited TLR induced activation of monocytes, and dose-dependently inhibited LPS-induced activation of monocyte-derived DCs comparable to human IL-10. By using blocking antibodies to either bovine IL-10 or the human IL-10 receptor it was demonstrated that inhibition of monocyte activation by bovine IL-10 was dependent on binding of bovine IL-10 to the human IL-10R. These data demonstrate that bovine IL-10 potently inhibits the activation of human myeloid cells in response to TLR activation. Bovine IL-10 present in dairy products may thus potentially contribute to the prevention of necrotizing enterocolitis and allergy, enhance mucosal tolerance induction and decrease intestinal inflammation and may therefore be applicable in infant foods and in immunomodulatory diets.

## Introduction

Dietary components are capable of modulating intestinal immune responses [1], [2]. Dairy products, including cow's milk, are widely consumed in Western societies and contain a wide range of immunoprotective factors such as immunoglobulins, lactoferrin, anti-microbial enzymes and cytokines. Bovine IL-10 was found to have an amino acid sequence identity of 76.8% with human IL-10, indicating that bovine IL-10 may exert functional effects on human immune cells [3], [4], [5], [6]. Therefore, bovine IL-10 present in dairy and dairy related products could potentially have immunomodulatory activity in the human consumer. Functional cross species activity of cytokines has been reported for chicken IFN-γ and turkey IL-2 [5], [6], and both porcine IL-2 and human IL-2 were reported to enhance proliferation of human, bovine, porcine and murine cells *in vitro* [3]. Also, human IL-10 is functionally active on a mouse mast cell line, but mouse IL-10 was not functionally active on a human B cell line [7]. Together these findings indicate that cytokines can be functionally active across species. The potential cross-species bioactivity of IL-10 depends mostly on the sequence identity of the IL-10 receptor (IL-10R) binding sites [4] and three dimensional structure of the proteins involved.

Biologically active IL-10 binds to the IL-10R, which is expressed on monocytes, macrophages, dendritic cells (DCs), NK cells, T cells and B cells. IL-10 is bound as a homodimer at two sites by both the IL-10R1 dimer and the IL-10R2 dimer, resulting in four IL-10/IL-10R interaction sites [8], [9], [10]. The IL-10R1 dimer binds the IL-10 molecule with high affinity; subsequently, this complex is recognized by the low affinity IL-10R2 dimer. IL-10 bound to IL-10R1 activates phosphorylation of Jak1 and Tyk2, which leads to Signal Transducer and Activator of Transcription 3 activation [11]. Signal Transducer and Activator of Transcription 3 translocates to the nucleus and activates Suppressor of Cytokine Signalling-3 [11], [12], resulting in suppression of MyD88 - NFκB activated TLR-inducible cytokines like IL-1β, IL-6 and TNF-α [13], [14], [15], [16], [17]. These cytokines are selectively inhibited by IL-10 in a dose-dependent manner [18], [19].

IL-10 is a potent cytokine and essential in controlling excessive immune responses to infections, thereby reducing immunopathology [20]. T cell dependent and T cell independent IgA class switching and production can be initiated by IL-10 in secondary immune organs and in the lamina propria [21], [22], [23]. IL-10 is involved in tolerance induction and immune regulation in both the innate and adaptive immune system. IL-10 can also inhibit homing of DC's to the draining lymph node [24], and IL-10 treated DCs can induce tolerance [25]. The maturation and activation induced expression of CD40, CD80 and CD86 by macrophages and DCs can be inhibited by IL-10 [26], [27], affecting the ability to stimulate T cells. Indeed, IL-10-exposed APCs fail to induce IFN-γ production by Th1 cells [28], [29].

IL-10 can directly regulate T cell responses and has been shown to be related to successful allergen immunotherapy [30], [31], [32]. IL-10 excreted by transfected *Lactococcus lactis* in the lumen of the intestine of mice can induce IL-10 production by cells of the Peyers patch and prevent allergic sensitization to food [33]. Next to this, in a neonatal rat model, decreased necrotising enterocolitis (NEC) correlated with increased in situ IL-10 production [34]. These findings show the potential significance of the presence of IL-10 in the intestine.

In this report, we investigated whether bovine IL-10 could exert functional activity on human monocytes and dendritic cells. Bioactive bovine IL-10 could potentially be used for the prevention of inflammatory diseases as NEC and allergy in infant nutrion, or in immunomodulating diets for patients suffering from intestinal inflammatory disorders. We show that bovine IL-10 is recognized by the human IL-10 receptor and dose-dependently inhibits cytokine production and surface marker expression during LPS induced DC maturation.

## Materials and Methods

### IL-10 sequence analysis

IL-10 sequences were obtained from the online databases of NCBI (http://www.ncbi.nlm.nih.gov/) and UniProt (http://www.uniprot.org/). Existing signal peptide data or signalP 3.0 (http://www.cbs.dtu.dk/services/SignalP/) were used to identify IL-10 signal peptides, which were removed before performing the sequence alignment. Sequence alignment was done in BioEdit (version 7.0.9.0) using ClustalW Multiple Alignment with default settings. Subsequently, the amino acid sequence identity was calculated using the sequence identity option in BioEdit. Accession numbers of the IL-10 sequences are: Human, UniProt, P22301; Bovine, UniProt, P43480, Epstein-Barr virus (EBV), UniProt, P03180; Rat, NCBI, EDM09836; Sheep, NCBI, CAA82546; Mouse, NCBI, AAI37845; Pig, NCBI, CAL29498 and Papiine herpesvirus 1 (PapHerp), NCBI, AAF23949.

### Three-dimensional modeling of bovine IL-10

The dimeric structure of human IL-10 (PDB entry: 1j7v, resolution 2.9 Å) was used as a template to model the dimeric bovine IL-10 protein using the program MODELLER (version 9v8 [35], [36]), which incorporates the CVFF force field [37]. Stereochemical quality of the homology models was assessed using the program PROCHECK [38]. Protein folding quality was verified using the program PROSAII [39], which independently evaluates the compatibility of each residue to its environment.

### PBMC isolation

Peripheral blood mononuclear cells (PBMCs) were diluted 1∶1 in IMDM (Gibco-BRL, Paisley, Scotland) and isolated by gradient centrifugation on Ficoll-Plaque PLUS (Amersham Biosciences, Uppsala, Sweden) for 5 minutes at 200 g and subsequently for 15 min. at 500 g (without brake at 20 °C). The PBMCs were harvested from the Ficoll layer, gently resuspended in IMDM and washed two or three times in IMDM.

### Monocyte isolation and stimulation

Monocytes from freshly isolated PBMCs were labeled with MicroBeads conjugated to mouse IgG2a monoclonal anti human CD14 antibodies (130-050-201, Myltenyi Biotec, Germany), and isolated using the quadroMACS (Myltenyi Biotec) according to the manufacturers descriptions. Briefly, cells were incubated with MicroBeads for 15 minutes at 4°C, washed with MACS buffer, centrifuged and resuspended in MACS buffer. The MACS columns were placed in the quadroMACS and rinsed. Subsequently, the cell suspension was added, rinsed and the columns removed from the quadroMACS; labeled cells were collected in a new tube by rinsing with MACS buffer and the supplied plunger. Purity of the CD14^+^ cell population was between 90 and 95%, as determined by flow cytometric analysis (FACS Canto II BD Biosciences, San Jose, CA, USA) by labeling the cells with mouse IgG2a anti human CD14 APC or an isotype control (clone M5E2, 555399 or 555576, BD Biosciences). Myltenyi Biotec indicated that clone M5E2 was not used on the microbeads used for isolation of the cells. After MACS sorting monocytes were resuspended in IMDM +1% Yssels medium [40] +1% penicillin and streptomycin (Gibco).

### Recombinant bovine IL-10

**Figure 1:**
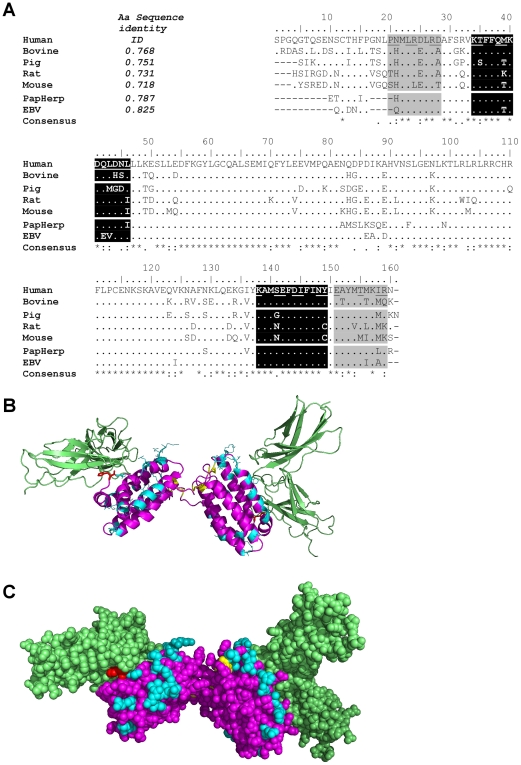
Comparison of human and bovine IL-10 in the human IL-10R. **A:** ClustalW sequence alignment of IL-10 from different species. IL-10 sequences were retrieved from the NCBI and Uniprot databases and analyzed for signal peptides (SignalP 3.0) which were removed from the sequences before performing the alignment (18 amino acids for human and bovine IL-10). At the top left the overall sequence identity is shown. In grey (Ib) and black (Ia) background shading the IL-10 receptor 1 binding sites are indicated as published by Josephson [8]. The underlined residues indicate >5 Å^2^ surface area in IL-10RA site I. At the bottom of each row the consensus (*) sequence is shown; “.” and “:” indicated homologous amino acids. **B:** Bovine IL-10 was modeled using human IL-10 in the human IL-10/IL-10R complex as template. The human IL-10R is shown in green and amino acid substitution between human and bovine IL-10 are depicted in cyan. Cysteine residues are colored yellow, the amino acid colored red (indicated with an arrow for one of the two IL-10 chains) is His 44, which is and amino acid substitution in close contact with the human Il-10R. **C:** The same model as in B, but displayed using spheres.

The recombinant bovine IL-10 was produced in house. Therefore the bovine IL-10 cDNA sequence encoding the mature part of the protein was cloned in pET15bGW essentially as described previously for rhinoceros IFN-γ [41]. Vector pET15bGW encodes for an N-terminal tag containing 6 histidine residues (his6-tag) under control of a T7 promoter. This tag enables purification of recombinant protein by immobilized metal affinity chromatography (IMAC). Protein production, purification by IMAC endotoxin removal and refolding was essentially performed as described previously for carp CXCL8 chemokines [42]. The sequence of recombinant bovine IL-10 used in the cell culture experiments is identical to the bovine IL-10 sequence showed in Figure 1 (NCBI database), as was confirmed by sequencing the IL-10 coding insert of the vector (Baseclear, Leiden, The Netherlands).

### IL-10R inhibition

Monocytes were stimulated with 1 µg/ml LPS (*S. enterica*, Sigma Aldrich, St Louis, USA), 2 µg/ml peptidoglycan (*S. aureus*, Fluka, Buchs Switzerland) and 2 µg/ml flagellin (*S. typhimurium*, InvivoGen, Toulouse, France). Human (200-10, Peprotech, London) or bovine IL-10 was used at a concentration of 10 ng/ml. To block the biological activity of recombinant bovine IL-10, 50 µg/ml total chicken IgY isolated from egg yolks of chicken immunized with recombinant bovine IL-10 was used. Antibodies isolated from egg yolks of non-immunized chicken (50 µg/ml) were used as a negative control. To block the human IL-10R, 5 µg/ml rat IgG2a anti human IL-10R (CDw210) (Clone 3F9, 556011, BD Biosciences) and an isotype control (554687, BD Biosciences) were used. Antibodies were added to the cells and incubated at 37°C and 5% CO_2_ for 30 minutes prior to addition of other stimuli. Monocytes were stimulated with different TLR ligands with or without bovine IL-10 for 24 hours at 37°C and 5% CO_2_. LPS and a widely used dose [12], [19] of 10 ng/ml human or bovine IL-10 were added and incubated for 24 hours at 37°C and 5% CO_2_. Cell culture supernatants were collected and stored at −80°C for later cytokine analyses.

### DC generation and stimulation

Monocytes for DC generation were purified from freshly isolated PBMCs by density centrifugation using Percoll (GE Healthcarespec. grav. 1.130±0.005 g/ml, <25 mOsm/kg H_2_0). Standard isotone Percoll was prepared by adding 1 part PBS to 9 parts Percoll and used to make three layers of different densities of Percoll (2.5 ml 60%, 5.0 ml 45% and 2.5 ml 34.2% in HBSS in a 15 ml tube). Cells (in HBSS +10% FBS) were gently added on top of the Percoll layers (max 40*10^6^ cells) and centrifuged at 18–22°C for 45 minutes at 1750 g without brake. Monocytes were collected and transferred to a new tube, centrifuged again (20 minutes at 1000 g) and washed two times with 1% FBS in PBS (Gibco, 16000-044). Isolated monocytes were cultured in FBS free CellGroD medium (CellGenix, 2005) with pen/strep (Gibco, 15140) at a concentration of 1*10^6^ cells/ml. 40 ng/ml recombinant human GM-CSF (Peprotech, 300-03) and 20 ng/ml recombinant human IL-4 (Peprotech, 200-04) were added and refreshed every second day. On day 6, expression of surface markers was analyzed using flow cytometry (FACS Calibur, BD Biosciences).

On day 6, DCs were stimulated with 50 ng/ml (or 1000 ng/ml, data not shown) LPS (*S. enterica*, Sigma Aldrich, St Louis, USA) for 48 hours in the absence or presence of IL-10. Cell free supernatants were collected and stored at −80°C until analysis. DCs were collected and cell surface marker expression was analyzed.

### Cell Surface marker analysis

DCs were stained with FITC or PE labeled mouse IgG1 antibodies anti human CD40 (FITC), CD80 (PE), CD83 (FITC), CD86 (FITC), CD1a (PE), MR (CD206, PE) or HLA-DR (PE) (555588, 557117, 556910, 555657, 555807, 555954, 555812, BD Biosciences) or isotype controls (PE 555749, FITC 555748, BD Biosciences), according to the manufacturers descriptions. Cells were incubated at room temperature for 30 minutes in the dark. Subsequently, cells were centrifuged, supernatant discarded, 200 µl FACS Buffer (BD Biosciences) added, and analyzed for surface marker expression by flow cytometric analysis.

### Human cytokine measurements

Human cytokine concentrations in cell culture supernatants were determined using the cytometric bead array flex sets (BD Biosciences) and incubations were performed in the recommended Protein Master Buffer Kit (BD Biosciences). Briefly, 50 µl supernatant was incubated with capture beads in 50 µl Capture Bead Diluent for one hour at room temperature. Next, PE Detection Reagent in 50 µl Detection Reagent Diluent was added and incubated for two hours at room temperature in the dark. For the standard curve, the provided lyophilized recombinant cytokines were used and assayed together with the samples. After incubation the samples were centrifuged, supernatant discarded and 200 µl Wash Buffer was added. The samples were analyzed by flow cytometric analysis (FACS Canto II, BD Biosciences).

### Bovine IL-10 ELISA

A bovine IL-10 specific capture ELISA was developed in house using an anti-bovine IL-10 specific monoclonal (MCA2110, AbD Serotec, Oxford, UK) as a capture antibody and biotinylated anti-bovine IL-10 IgY as a detecting antibody. The anti-IL-10 IgY had been affinity purified against in house produced recombinant bovine IL-10 coupled to CNBr-activated Sepharose 4B from eggs of chickens that had been immunized repeatedly with recombinant bovine IL-10.

### Statistical analysis

Statistical analysis was performed with PASW Statistics SPSS version 17.0.3. Cytokine production levels upon stimulation with bacterial ligands vary greatly between donors. Therefore we tested the inhibitory capacity within the donor. We performed a generalized linear model after logarithmic transformation of the cytokine data: cytokine production level  =  donor (stimulus * inhibitor). Stimulus (LPS, PGN or Flagellin) and inhibitor (whether IL-10 was added or not) were included as fixed factors (this will compare groups) and the interaction term (*) tests the effect of the inhibitor in relation to the stimulus used.

Statistical analysis of the dose-dependent modulation of DC surface marker expression levels by bovine IL-10 was performed using a one by one correlation model: surface marker expression level or cytokine concentration  =  bovine IL-10 concentration.

The bioactivity between bovine and human IL-10 was compared using a univariate general linear model: cytokine production  =  IL-10 source (bovine or human) * IL-10 concentration. Low p-values would indicate a difference between human and bovine IL-10.

## Results

### Identification of human IL-10R binding sites in bovine IL-10

In order to make a more detailed comparison possible of potential interactions between bovine IL-10 and human IL-10R, IL-10 sequences of different species were aligned and the IL-10R binding sites indicated (Figure 1). IL-10 sequences were obtained from the NCBI and Uniprot online databases, and SignalP 3.0 was used to identify signal peptides. Next, signal peptides were removed from the sequences before performing the ClustalW Multiple Alignment. Data on the IL-10R binding sites (obtained from Josephson [8]), were in line with the receptor binding sites reported by Reineke [9]. The IL-10R binding sites in IL-10, as published by Josephson, are indicated in Figure 1A, with black (Ia) and grey (Ib) background colors. The interactions between IL-10 and the IL-10R are largely determined by residues of IL-10 in close contact with the receptor. Therefore, residues burying a surface area of more than 5 Å^2^ into human IL-10R1 [8] are underlined in Figure 1A, allowing evaluation of potential consequences of sequence differences between human and bovine IL-10 in the human IL-10R binding sites.

Human and bovine IL-10 were found to have an amino acid sequence identity of 76.8% (Figure 1A). Twenty-nine percent (11 out of 38) of the amino acid substitutions are present in the first 20 amino acids of the IL-10 sequence. The IL-10R binding site Ia (amino acid residues 138–149) is identical for human and bovine IL-10. Divided over the three other IL-10R binding sites, bovine IL-10 contained nine amino acid substitutions compared to human IL-10 (summarized in Table 1). Seven of the nine different residues bury more than 5 Å^2^ in the IL-10R binding site and therefore are expected to contribute to the binding of IL-10 to the IL-10R. Three amino acid substitutions had a different polarity, and different hydrophobicity indexes. These three non-homologous substitutions occurred in the Ib IL-10R binding sites and two of them buried more than 5 Å^2^ in the IL-10R binding site. Six of the nine amino acid substitutions had similar polarity and none had opposite (positive versus negative) polarity.

**Table 1:** Summary of bovine IL-10 amino acid substitutions in human IL-10R binding sites.

Binding site(residues)	Ib(20–28)	Ia(34–46)	Ia(138–149)	Ib(151–159)	Total
No. residues	9	13	12	9	43
Substitutions	3	2	0	4	9 (21%)
*- Homologous*	2	2	-	3	7 (78%)
Substitutions >5 Å^2^	2	2	-	3	7
*- Homologous*	1	2	-	2	5 (71%)

Data are obtained from a ClustalW Multiple alignment as shown in Figure 1A. Of the amino acid substitutions and the substitution burying >5 Å^2^ in the human IL-10R [9] the number of homologous amino acid substitutions is indicated.

To support the linear peptide analysis, a 3D model of bovine IL-10 bound to the human IL-10R was built, based on the available crystal structure of the human IL-10/IL-10R complex (Figure 1B). No structural differences apart from some side chain orientations are observed between the model of bovine IL-10 and the crystal structure of human IL-10. The majority of amino acid substitutions between human and bovine IL-10 are located at the surface of the IL-10 molecule. The IL-10R binding site of IL-10 does not contain any amino acid substitutions, except for one histidine residue (His 44) substitution. Analysis of the 3D model showed that this histidine is located in a pocket of the receptor (Figure 1C) and is not likely to impair IL-10R binding.

### Bovine IL-10 exerts functional effects on human monocytes through binding to the IL-10R

In order to investigate the bioactivity of bovine IL-10 on the human immune system, we tested the effect of bovine IL-10 on freshly isolated monocytes from three healthy donors. These freshly isolated monocytes were stimulated with different bacterial ligands: peptidoglycan (PGN), flagellin and lipopolysaccharide (LPS) in the presence or absence of bovine IL-10.

**Figure 2:**
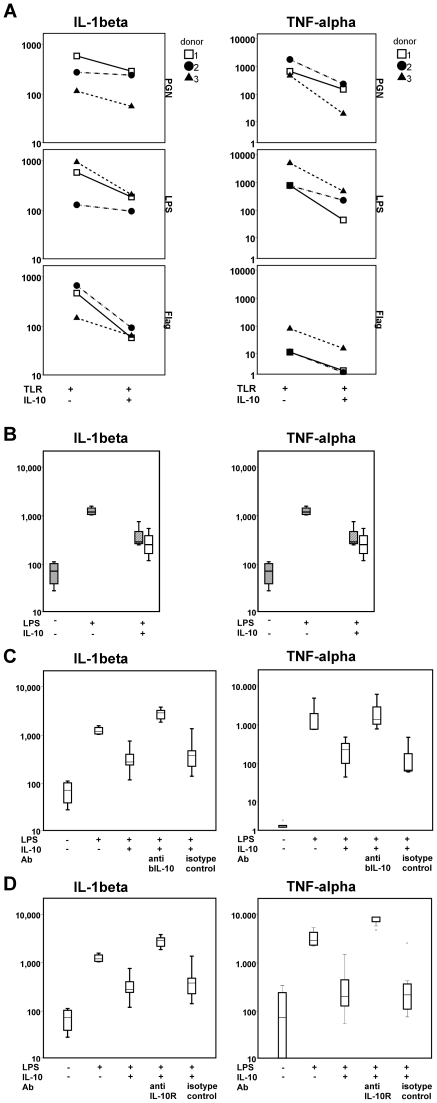
Bovine IL-10 can regulate TLR ligand induced cytokine production by monocytes by binding the IL-10R. Figures 2B-2D are box plots, showing the median (black horizontal bar), 50% data range (box) and 99% data range (error bars). On the x-axes is the addition indicated (+) of TLR stimuli (panel A), or 10 ng/ml LPS (B–D), 10 ng/ml IL-10 (A-D), bovine IL-10 or human IL-10R blocking antibodies (anti bIL-10 or IL-10R) or isotype control (C–D). **A**: Freshly isolated monocytes were stimulated for 24 hours with different ligands (lipopolysaccharide (LPS); Flagellin (Flag); peptidoglycan (PGN)) with or without recombinant bovine IL-10. IL-1β (p<0.001) and TNF-α (p<0.001) were significantly inhibited by the three bacterial ligands tested. Data (pg/ml) is shown for three different donors. **B:** Human monocytes were stimulated with LPS and either human (white box) or bovine (hatched box) IL-10 was added to compare the inhibitory capacity of bovine IL-10 with human IL-10. **C:** To confirm that the response is specifically inhibited by bovine IL-10 a blocking antibody and an isotope control were pre-incubated with bovine IL-10 and LPS and subsequently added to the monocytes. IL-1β and TNF-α production (pg/ml) is shown of three different donors. **D:** In order to proof that the bioactivity of bovine IL-10 is mediated through the IL-10R, monocytes were pre-incubated with IL-10R blocking antibodies and subsequently stimulated with LPS and bovine or human IL-10. Data is shown of 3 different donors.

Monocyte cell cultures were 90–95% pure, as determined by flow cytometry for CD14 (data not shown). Addition of recombinant bovine IL-10 significantly inhibited PGN, flagellin and LPS induced IL-1β (p<0.001) and TNF-α (p<0.001) production by monocytes as tested on three different donors (Figure 2A). We suggest that bovine IL-10 potently inhibits TLR induced activation. The 5-fold inhibition of TNF-α and IL-1β production by monocytes was comparable between bovine and human IL-10 (Figure 2B and Table 2).

**Table 2:** Percentage inhibition of DC and monocyte activation of human and bovine IL-10.

		DC								monocytes			
		IL-12		Tnf-α		CD83		CD86		IL-1β		TNF-α	
		bo	hu	bo	hu	bo	hu	bo	hu	bo	hu	bo	Hu
**IL-10 (ng/ml)**	**0**	100	100	100	100	100	100	100	100	100	100	100	100
	**0.25**	102.9	52.5	143.9	92.1	130.5	128.7	106.3	105.6				
	**0.75**	30.8	49.0	88.0	74.7	125.5	114.4	83.4	99.2				
	**2.2**	28.0	16.3	55.2	25.1	125.2	60.6	99.3	83.7				
	**6.7**	8.9	6.4	13.1	4.6	90.9	72.0	67.9	62.2				
	**20**	1.5	2.1	0.9	1.3	38.2	26.2	51.0	47.3				
	**10**									30.3	23.0	14.2	6.1

Average percentage (3 donors, Figure 4) of IL-12 and TNF-α production and CD83 and CD86 expression by DCs and IL-1β and TNF-α production by monocytes (4 donors, Figure 2B) is shown compared to cells only stimulated with LPS (DC's 10 ng/ml, monocytes 1 µg/ml). Similar findings showing the similarity in inhibitory capacity of bovine and human IL-10 were obtained for expression of CD40, CD80, CD1a, MR and HLA-DR.

Additional experiments were performed to confirm that inhibition of TLR-induced cytokine production by monocytes was specifically caused by bovine IL-10. Therefore, IL-10 blocking antibodies or isotype control antibodies were pre-incubated with bovine IL-10 and LPS, and subsequently added to the monocytes. Addition of IL-10 blocking antibodies in combination with bovine IL-10 completely restored the IL-1β and TNF-α production by LPS stimulated monocytes; which was not observed when the isotype control was used (Figure 2C).

The comparison of the sequence of bovine IL-10 with the IL-10R binding sites on the sequence of human IL-10 (Figure 1), confirmed that bovine IL-10 could potentially bind to the human IL-10R. To investigate whether the functional effect of bovine IL-10 is indeed through the binding of bovine IL-10 to the human IL-10R, additional experiments were performed with an human IL-10R blocking antibody (anti human CDw210). PBMC derived monocytes from three different donors were isolated and pre-incubated with the IL-10R blocking antibody or isotype control. Following this incubation, LPS and bovine IL-10 were added. Blocking the IL-10R completely restored the IL-1β and TNF-α production of LPS stimulated monocytes, proving that bovine IL-10 binds to the human IL-10R (Figure 2D). Addition of the isotype control resulted in TNF-α and IL-1β production levels similar to IL-10 inhibited monocytes. The results obtained with the IL-10R blocking antibody showed a similar inhibition as the results from the bovine IL-10 blocking antibody.

These results clearly demonstrate that bovine IL-10 inhibits TLR induced cytokine production by human monocytes, through binding to the human IL-10R.

### Bovine IL-10 inhibits LPS-induced DC activation

After testing the bioactivity of bovine IL-10 on human monocytes, experiments with DCs were performed to test if bovine IL-10 is bioactive on other antigen presenting myeloid cells that are potent T helper cell inducers. Freshly isolated monocytes were differentiated into immature DCs under the influence of human IL-4 and GM-CSF. These immature DCs from three different donors were maturated with 50 ng/ml LPS in the presence of several concentrations of bovine IL-10. The LPS-induced cytokine production and cell surface marker expression were characterized by flow cytometric analysis.

**Figure 3:**
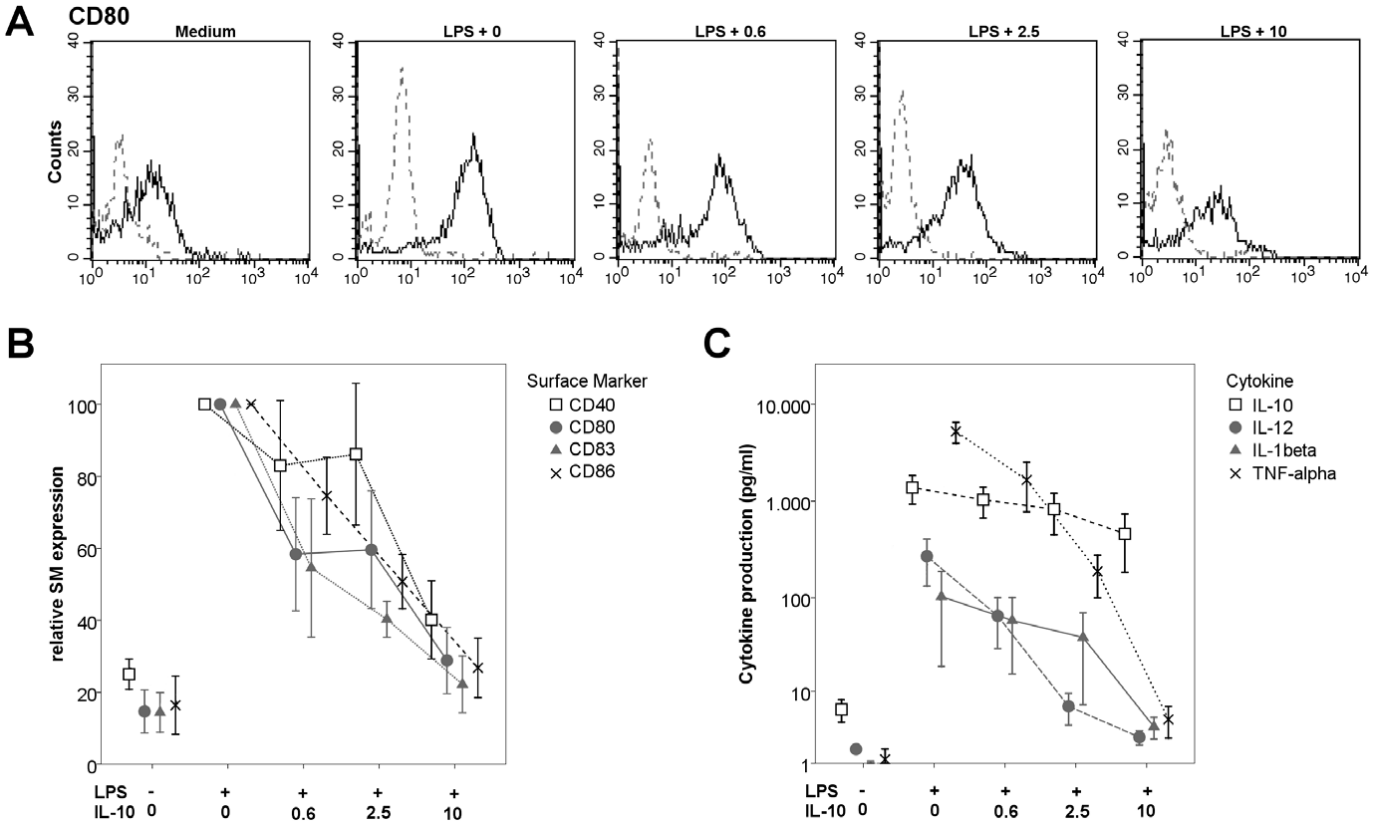
Bovine IL-10 dose-dependently inhibits DC surface marker expression and cytokine production. **A:** Typical example of raw data of flow cytometric analysis. Data shown is CD80 expression during LPS induced DC maturation of one donor. The solid line indicates CD80 staining and the dashed line the isotype control. On top of the graphs is indicated whether medium, LPS or LPS plus different doses of IL-10 (ng/ml) were used. **B:** Bovine IL-10 dose-dependently modulates DC surface marker expression (CD83, p = 0.002; CD40, p = 0.030; CD80, p = 0.018) during LPS induced maturation. Relative values are shown from three different donors. Mean fluorescent intensities were divided by the isotype control and expressed relative to the positive control (LPS, without IL-10), which was set at 100%. **C:** Recombinant bovine IL-10 dose dependently modulates the production of cytokines by human DC's during LPS induced maturation (IL-12, p = <0.001; TNF-α, p<0.001; IL-1β, p<0.001, IL-10, p<0.001). Raw data is shown from three different donors tested. For each cytokine, a negative control (no LPS, no IL-10) is shown at the left. Data is shown in panels B and C are from three different donors, error bars indicate standard error. On the x-axes the addition of LPS and bovine IL-10 (ng/ml) is indicated.

A typical example of raw flow cytometry data is shown in Figure 3A (CD80). Maturation marker CD83 and activation markers CD40, CD80 and CD86 were up-regulated after stimulation with LPS. Expression of CD83 (p = 0.006), CD40 (p = 0.030), CD80 (p = 0.018) and CD86 (p = 0.012) were dose-dependently down-regulated by bovine IL-10 (Figure 3B). Expression of mannose receptor (MR) (p = 0.002) was up-regulated with increasing doses of bovine IL-10. The expression of HLA-DR was not influenced by bovine IL-10 or LPS. The expression of CD1a was inhibited after stimulation with LPS, and not affected by bovine IL-10 (data not shown). Addition of bovine IL-10 alone did not affect any of the analyzed surface marker expression levels (data not shown).

Cell culture supernatant of LPS (50 ng/ml) stimulated DCs were analyzed for IL-12p70, TNF-α, IL-1β and IL-10 levels. Bovine IL-10 dose-dependently down-regulated the production of IL-12p70 (p<0.001), TNF-α (p<0.001), IL-1β (p<0.001) and IL-10 (p = 0.001) as shown in Figure 3C. Similar results were obtained for DC's stimulated with 1000 ng/ml LPS (data not shown). On both, DCs and monocytes, 10 ng/ml bovine IL-10 could almost completely inhibit TLR dependent activation.

These data clearly show that bovine IL-10 dose-dependently inhibits LPS induced surface marker expression and cytokine production by DCs.

### Bovine and human IL-10 are equally effective in inhibiting LPS-induced DC activation

As bovine IL-10 is able to dose-dependently modulate DC responses, a comparison of the bioactivity of bovine IL-10 and human IL-10 was made, to verify if bovine IL-10 is equally potent as human IL-10 on human dendritic cells. Compared to the previous experiment, a wider range of IL-10 concentrations was used in combination with 10 ng/ml LPS. Expression of CD40, CD80, CD83, CD86, HLA-DR, CD1a, MR and TNF-α and IL-12p70 production was determined for three different donors.

**Figure 4:**
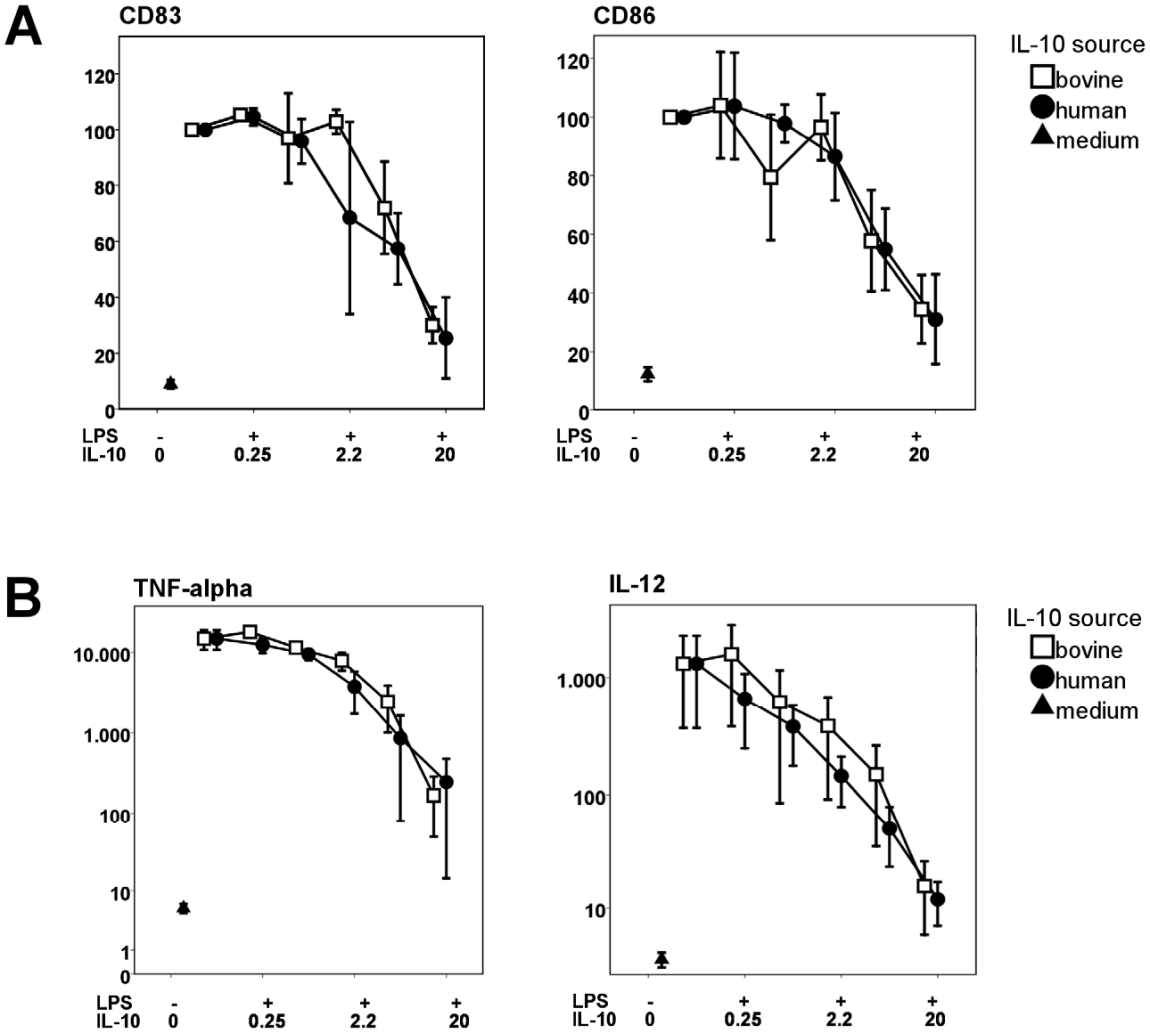
Dose-dependent inhibition during LPS-induced DC maturation is comparable for human and bovine IL-10. Data shown are from 3 different donors tested, error bars indicate standard error. **A:** Dose dependent inhibition of CD83 (p = 0.753) and CD86 (p = 0.936) by human and bovine IL-10. Data were divided by the isotype control and expressed relative to the positive control of only LPS, which was set at 100%. **B:** Dose dependent inhibition of TNF-α (p = 0.916) and IL-12p70 (p = 0.962) production by human and bovine IL-10, shown in pg/ml.

Human and bovine IL-10 were both equally potent in inhibiting LPS induced CD83 (linear regression of the difference between human and bovine IL-10: p = 0.753 and CD86: p = 0.936) expression by LPS stimulated DCs (Figure 4A and Table 2). Also expression of CD40 (p = 0.995), CD80 (p = 0.971), HLA-DR (p = 0.841), CD1a (p = 0.873) and MR (p = 0.881) was not differentially modulated between human and bovine IL-10 (data not shown). Likewise, the TNF-α (p = 0.916) and IL-12p70 (p = 0.962) production was equally modulated by human and bovine IL-10 (Figure 4B). From Table 2 it appears that bovine and human IL-10 show similar inhibitory capacity for all parameters tested.

Based on these data, we conclude that bovine and human IL-10 are equally potent in inhibiting LPS induced DC activation.

### Bioavailability of bovine IL-10

As applications of bovine IL-10 in humans would depend on the bioavailability we analyzed milk and colostrum samples for IL-10 levels by ELISA and performed an in vitro assay to assess the survival of IL-10 in the upper digestive tract.

In colostrum samples, a range of 150–3000 pg/ml of IL-10 was detected in 56 samples from 10 different cows, but in commercially available milk no significant IL-10 levels were detectable (data not shown).

To evaluate the survival of bovine IL-10 in the human upper digestive tract, IL-10 was incubated in an electrolyte solution containing pepsin. IL-10 was dissolved in a protein matrix and incubated for 1 (adults) or 2 (infants) hours at different pH (pH 3 for adults, pH 4 for infants) to resemble respectively the infants and adult stomach. After this incubation 50–60 percent of the IL-10 was still detectable under the conditions of an infant's stomach and 20% for adults (data not shown).

## Discussion

Dairy products form an important part of the Western diet, and are a potential source of immunomodulatory ingredients. Bovine milk contains cytokines like IL-10 that can have functional effects on immunological structures in the gut mucosa. IL-10 can exert bioactivity when present in the lumen of the gut [33] and therefore, consumption of IL-10 may potentially modulate innate immune responses in the intestine. The data presented here demonstrate that bovine IL-10 is able to modulate TLR induced cytokine production by human monocytes. Bovine IL-10 binds to the human IL-10R and is able to dose-dependently modulate LPS-induced DC activation, similar to human IL-10. This is in line with the results of a detailed analysis of the cytokine receptor binding sites, and with 3D modeling of the human IL-10R/bovine IL-10 complex.

The recombinant bovine IL-10 used here exerted similar inhibitory capacity and dose-dependency on dendritic cells as human IL-10. The effects noted with human IL-10 are in line with IL-10 studies published before [13], [15]. These results demonstrate that the amino acid substitutions between human and bovine IL-10 do not affect the interaction between bovine IL-10 and the human IL-10R.

Other amino acid substitutions in IL-10 may however decrease IL-10 functionality. An extensive genomic analysis of the IL-10 gene and flanking regions of 94 Ulcerative colitis and 94 Crohn disease patients revealed polymorphisms in the IL-10 coding gene, of which one (R159) was in the receptor binding site, as reported by Josephson [8], [43]. However, it has not been studied in detail if this polymorphism indeed affects IL-10 – IL-10R interactions in colitis patients.

As far as we know, the IL-10R expression and signal transduction is similar for monocytes and DCs with regard to TLR-dependent activation and cytokine production [11], [12], [44]. Indeed, our results show that cytokine production by monocytes and DCs could be equally well modulated by bovine IL-10. Involvement of the human IL-10R in the bioactivity of bovine IL-10 implies that all cell types like T cells, B cells and NK cells expressing the IL-10R are probably also modulated by bovine IL-10.

Monocytes and DCs are antigen presenting cells (APC) that are involved in downstream activation of T cells. The modulation by bovine IL-10 of TLR-induced cytokine responses and surface marker expression on DCs will in turn affect their ability to activate CD4^+^ T helper cells. T helper cells need co-stimulation of CD28 by CD80 (B7-1) or CD86 (B7-2). Bovine IL-10 reduced the upregulation of CD80 and CD86 by human DC's and therefore is likely to reduce T helper cell activation [45]. In addition, bovine IL-10 also modulated IL-12 production, which affects Th1 versus Th2 skewing. As shown in figure 3C, this downregulation of cytokines was more prominent for proinflammatory cytokines (IL-1β, TNF-α, and IL-12), suggesting that bovine IL-10 may dampen Th1 development. The prevention of upregulation of CD40 by bovine IL-10 prevents activation of DCs by CD40L from activated T cells and will reduce the activation of naive T cells by DCs. Moreover, bovine IL-10 can bind to the IL-10Rs expressed on T cells and directly inhibit their activation [45], [46], [47].

IL-10 and TGF-β both play a role in tolerance induction [48], [49] and have complimentary suppressive activity [50]. The presence of intact IL-10 could enhance the success of allergen immunotherapy, as the presence of IL-10 with an antigen at mucosal sites induces tolerance to that allergen [33]. The presence of IL-10 in the lumen resulted in elevated levels of IL-10 producing cells in the Peyers patch [33]. IL-10 in the lumen may be detected by DCs that protrude their dendrites through the epithelial cell layer to sample antigens [41], [51] and mouse epithelial cells were proven to express the IL-10R [52]. In individuals tolerant to mucosal antigens, more IL-10 dependent regulatory CD4^+^ cells are present in the periphery and sublingual allergen immunotherapy induced TGF-β and IL-10 dependent induction of T regulatory cells [50]. CD4^+^ cells activated in the presence of IL-10 become anergic and can acquire antigen specific regulatory activities [30], [53].

IL-10 is essential to develop healthy intestinal immunity [54]. That IL-10 present in the lumen can exert biological activity is shown in a mouse model of autoimmune encephalomyelitis and diabetes where orally administered IL-10 effectively enhanced tolerance induction [33]. In addition oral administration of TGF-β has proven to be functionally active in the intestine when using a mouse model [55]. Likewise, results from our in vitro experiment suggest that bovine IL-10 is able to survive and therefore may be bioactive, particularly in the upper digestive tract of infants. Like in bovine colostrum, IL-10 is present in human breast milk in comparable concentrations ranging from 40 pg/ml till 3 ng/ml [56], [57], [58]. As infant nutrition is based on resembling breast milk as much as possible, bovine IL-10 could be considered as a replacement for the IL-10 present in human breast milk.

Interestingly, the development of necrotizing enterocilitis (NEC) in premature infants is associated with lower levels of IL-10 in breast milk [59]. In addition, IL-10 knockout mice develop a phenotype resembling NEC [54]. Necrotizing enterocolitis induced in rats can be reversed by human breast milk as well as IL-10 [34], [60], and increased cytoplasmic IL-10 levels in epithelial cells in rats correlated with protection to NEC [34]. Moreover, injection of rats with induced NEC with anti TNF-α antibodies reduced incidence and severity [61]. IL-10 might have similar local effects as IL-10 is a potent regulator of TNF-α production.

From a meta-analysis of the literature, breastfeeding was concluded to be associated with a reduced risk for the development of allergy in the first decade of life [62]. The effect of breastfeeding may increase with duration of breastfeeding [62]. Next to IL-10, TGF-β is present in both human and bovine milk. TGF-β1 and TGF-β2 amino acid sequence identity between cow's and human is 95% respectively 98% after removing the signal peptide, and 100% in their cleaved form (own analysis, data not shown), and therefore are likely to be bioactive in humans. Prolonged application of a low dose of a cytokine could exert functional activity, as has been published for IL-2 [63]. Likewise, application of bovine IL-10 or TFG-β in infant nutrition over a long period may contribute to the prevention of allergy. Administration of bovine IL-10 is mainly expected to be advantageous for individuals with inflammatory diseases, but not for immune-compromised individuals as IL-10 may further inhibit immune activation. Additional administration of IL-10 should be done carefully. This requires further study.

Although several in vivo studies have shown IL-10 bioactivity after oral administration, a potential drawback of applying bovine IL-10 in infant nutrition is that to exert its biological effect in the intestines, bovine IL-10 administered through the diet has to pass the acidity and digestive enzymes of the human upper digestive tract. Another challenge would be to adapt current food processing procedures that currently affect the biological activity of IL-10 [56]. We do not expect that anti bovine IL-10 antibodies will appear when bovine is administered orally, and since bovine IL-10 is bioactive it can inhibit immune cell activation which is a prerequisite for the development of specific antibodies.

We conclude that bovine IL-10 exerts biological activity comparable to human IL-10 on human monocytes and DC's. These findings may have implications for the induction of immune tolerance in the intestinal mucosa in patients with intestinal inflammatory diseases, and for the prevention of NEC and allergy in infants.
